# Australian native flower colours: Does nectar reward drive bee pollinator flower preferences?

**DOI:** 10.1371/journal.pone.0226469

**Published:** 2020-06-11

**Authors:** Mani Shrestha, Jair E. Garcia, Martin Burd, Adrian G. Dyer

**Affiliations:** 1 Bio-Inspired Digital Lab (BIDS-Lab), Schools of Media and Communication, RMIT University, Melbourne, Australia; 2 School of Biological Sciences, Monash University, Melbourne, Australia; Indian Institute of Science, INDIA

## Abstract

Colour is an important signal that flowering plants use to attract insect pollinators like bees. Previous research in Germany has shown that nectar volume is higher for flower colours that are innately preferred by European bees, suggesting an important link between colour signals, bee preferences and floral rewards. In Australia, flower colour signals have evolved in parallel to the Northern hemisphere to enable easy discrimination and detection by the phylogenetically ancient trichromatic visual system of bees, and native Australian bees also possess similar innate colour preferences to European bees. We measured 59 spectral signatures from flowers present at two preserved native habitats in South Eastern Australia and tested whether there were any significant differences in the frequency of flowers presenting higher nectar rewards depending upon the colour category of the flower signals, as perceived by bees. We also tested if there was a significant correlation between chromatic contrast and the frequency of flowers presenting higher nectar rewards. For the entire sample, and for subsets excluding species in the Asteraceae and Orchidaceae, we found no significant difference among colour categories in the frequency of high nectar reward. This suggests that whilst such relationships between flower colour signals and nectar volume rewards have been observed at a field site in Germany, the effect is likely to be specific at a community level rather than a broad general principle that has resulted in the common signalling of bee flower colours around the world.

## Introduction

Many floral traits play a role in the reproduction of animal-pollinated angiosperms [1–5]. Colour is one of the most important signals used by flowering plants to communicate to their pollinators [6–10]. Flowers typically present nutritional rewards like nectar to entice floral visitors [6,11–15], and nectar is a reward that can promote learning and neural changes in a bee’s visual system [16]. What relationship between floral colour and nectar should evolve in plants? Whilst nectar has been well studied in flowering plants [15,17–20], the question of a potential relationship has been rarely considered because many animals have very different colour vision to humans [21]. Thus, colour is not an unambiguous trait, and to test colour as a factor in a biologically meaningful way it is necessary to map how relevant pollinators like bees perceive and use colour information.

Both male and female fitness of plants should often benefit from pollinator visits, especially given widespread pollen-limitation of seed set [22]. Such fitness benefits for plants will be especially strong with flower visitors like bees that tend to be ‘flower constant,’ that is, to use colour signals to repeatedly visit flowers of the same plant species [23] which likely increases conspecific pollen transfer. In turn, the ability of pollinators to assess which floral colour signals are more reliable predictors of nutritional rewards will affect the foraging success of individuals [24] and thus the subsequent success of bee colonies [14,25–27]. It has even been suggested that innate color preferences of naïve bees and their learning speeds for different colors correlate with typical nectar reward levels in flowers of different colors [28]. Such innate colour preferences would, however, be susceptible to exploitation by plant species that offer low rewards but display preferred floral colors. Thus, it seems unlikely that an association between floral color and nectar reward (an evolutionarily stable honest signal) could be the ecological basis for innate color preferences by bees.

Colour vision requires multiple photoreceptors with different sensitivities [29]. The spectral sensitivities of photoreceptors in many bee species have been empirically determined, showing that the trichromatic colour vision of bees is highly conserved and predates the evolution of flowers [30]. To yield colour information, photoreceptor signals have to be antagonistically processed in a brain [21]. Such colour opponent mechanisms in bees have been empirically recorded [31–33]. Knowing this information enables the construction of a colour space to represents colour information. A hexagonal colour model is an opponent colour space that accurately represents the visual capabilities of trichromatic bees [33].

**Fig 1:**
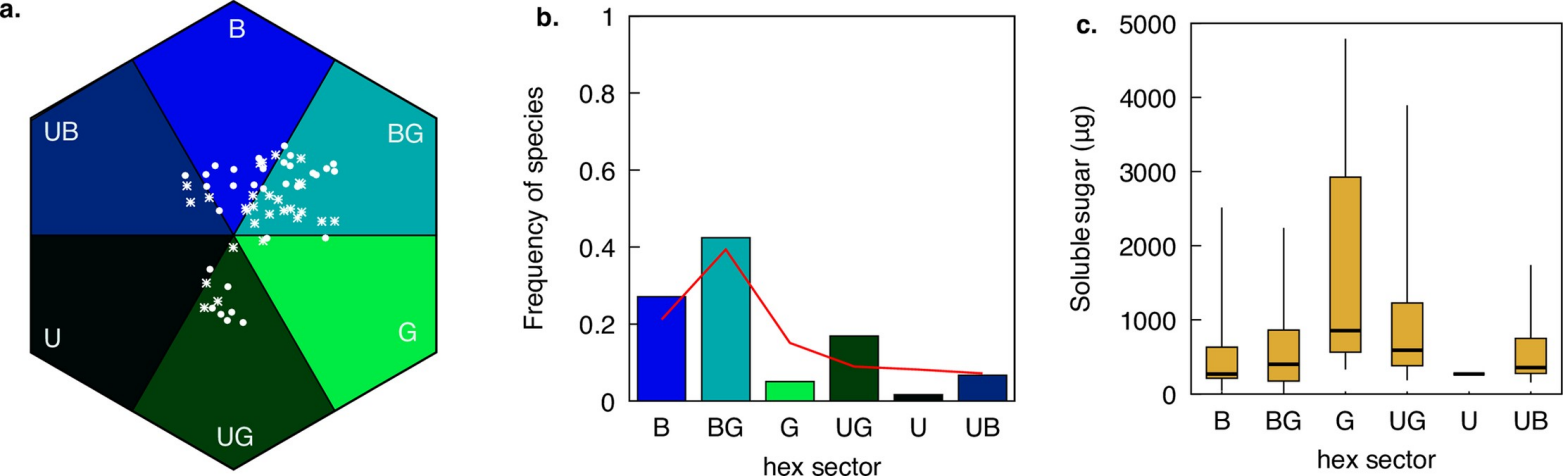
Flower colour and nectar in Australian native plant flowers. a). Distribution of 59 flowering plant species in hexagon colour space: non-orchids (•) and orchids (*). b). Frequency of sampled species classified on each of the six hexagon categories along with the corresponding pattern (red line) of plant species taken from the surveys of plant communities in Germany, Australia, and Nepal [40,43,44,45]. c). Plant flower soluble sugars by colour category; thick lines represent medians, boxes represent the 25% and 75% interquartile ranges, and thin vertical bars represent 2.5 and 97.5% quantiles of the data distribution. Names of the different hexagon sectors are abbreviated: BLUE (B), BLUE-GREEN (BG). GREEN (G), UV-GREEN (UG), UV (U), and UV-BLUE (UB) as described by [40].

Interestingly, both honeybees and bumblebees show similar distinct preferences for short wavelength ‘blue’ stimuli that frequently have loci in the UV-BLUE, BLUE and/or BLUE-GREEN sectors of bee colour space [28,34–39]. (In the current manuscript we use capitals (e.g. BLUE) to convey a region in bee colour space (e.g. see Fig 1A), and ‘blue’ to refer to how humans typically describe colour stimuli, following the convention proposed by [40]). It has also been recently shown that native bees in Australia show a significant colour preference for stimuli in the BLUE and BLUE-GREEN regions of bee colour space [41,42]. These potentially common bee colour preferences provide a plausible explanation for why bee-pollinated flowers from several fields sites around the world frequently share similar distributions in colour space [43]. For bee colour preferences to have evolved due to an association between floral colour and nectar reward [28], the association would have to be ancient and ubiquitous.

Floral colour distributions in natural plant communities have recently been documented from a geographically wide array of sites [3,46–53] including several recent studies that use sophisticated modeling of pollinator colour vision [43,53–55]. Where bees are present in both the Northern [3,44,56] and Southern [45–58] Hemispheres, flowers tend to have evolved colour signals that are efficiently processed by bee trichromatic vision. In Australia it has been observed at both a continental level [45,58] and a local community level [43,55] that flower colours share a very similar distribution in colour space to flower colour distributions in the Northern Hemisphere where bees with a blue preference are the dominant pollinator. Evolutionary change in floral colour appears not to be strongly limited by phylogenetic constraints [43,44,48,54,58].

In a study at a field site near Berlin, Germany, flowers were most frequently found to be in the UV-BLUE region of colour space and 80% of these flowers were reported to have high nectar rewards, while no other colour category contained more than 50% high-rewarding flowers [28]. In the current study, we seek to understand if a herbaceous community in Australia also shows a significant association between bee-perceived floral colours and nectar rewards that is consistent with the pattern found in Germany [28], and thus whether this association might be an important driver behind the evolution of bee colour preferences.

## Materials and methods

### Ethics

The study involved no animals, and this did not require ethics. Plant samples were collected with permit number 10005294, Parks Victoria and the Department of Sustainability and Environment 2009–2013.

### Sites and data collection

We collected data from two natural communities in central Victoria, Australia: Baluk Willam Flora Reserve (37°55′32″S, 145°20′45″E), 40 km south east of Melbourne, and Boomers Reserve (37°37′39″S, 145°15′21″E), approximately 35 km north east of Melbourne. Both sites are *Eucalyptus* woodland with well-developed shrub and herb layers containing a high diversity of orchid species. These communities have been protected to maintain native vegetation. Flowers were sampled from March 2010 to May 2011. Species were identified with the aid of several published identification keys to the local flora [59–66]. A list of species included in this study is given in online S1 Appendix.

### Nectar measurement

We used the floral nectar data of 59 bee pollinated flowering plant species in our analysis. Nectar collection and measurement methods are detailed in [55]. Briefly, newly opened flowers were placed in pollinator exclusion nets for 24 hours to allow nectar accumulation. Flowers were then excised, and soluble sugars were extracted by immersing whole flowers in known volume of distilled water followed by an acid treatment to reduce all sugars into hexoses. Subsequently, we measured the concentration of soluble sugars using standard spectrophotometric methods and back calculated the total sugar content of each flower [67]. Quantity of sucrose-equivalent present in each flowering plant species is listed in online supplementary [55] and S1 Appendix.

### Colour measurement

Reflectance spectra from 300 to 700 nm wavelength were measured on a minimum of three flowers for each species using an Ocean Optics spectrophotometer (Dunedin, Florida, USA) with a PX-2 pulsed xenon light source. A UV-reflecting white standard was used to calibrate the spectrophotometer. Spectra from multiple flowers were averaged within each species. For flowers with multiple colours, such as areas with and without a UV component, we obtained reflectance spectra of the two colours covering the largest surface area of the flower.

### Colour space representation

Floral reflectance spectra were converted to positions in a hexagonal colour space, a two-dimensional representation of the excitation levels of the three different classes of photoreceptors in a hymenopteran insect’s visual system [68]. This model is widely accepted as a representation of bee trichromatic vision in comparative studies of flower evolution [3,28,38,40,41,43,51,54,56,45,69,70]. The exact photoreceptor sensitivities of native Australian bees are currently unknown, but relying on the phylogenetic conservation of spectral sensitivity peaks of hymenopteran photoreceptors [30], we use a general hymenopteran visual model based on a vitamin A1 template for photopigments [71] with sensitivity peaks at 350 nm (ULTRAVIOLET: UV), 440 nm (BLUE: B) and 540 nm (GREEN: G) (cf. [45]). We calculated the relative probability of photon capture (*P*) by each of the UV, B, and G photoreceptors by numerically integrating the product of the spectral sensitivity function of each one of the (*i* = 3) photoreceptors *S*_*i*_(*λ*), the diffuse spectral reflectance of each flower *I*(*λ*) and the spectral distribution of the ambient illumination *D*(*λ*) expressed as photon flux [72]. All spectral functions were expressed from 300 to 650 nm at 10 nm steps:

$$P_{i}=R_{i}\int_{300}^{650}S_{i}(\lambda )I(\lambda )D(\lambda )d\lambda .$$ (1)

The coefficient *R*_*i*_ in Eq 1 represents von Kries adaptation and is used to normalize each of the photoreceptors to the illumination reflected from the background [68]:

$$R_{i}=1/\int_{300}^{650}S_{i}(\lambda )I_{B}(\lambda )D(\lambda )d\lambda ,$$ (2)

where *I*_B_ (λ) is the spectral reflectance of the background. We used the average reflectance from 20 species of *Eucalyptus* (Average Green Leaf) as background reflectance (*I*_*B*_(λ)) for our calculations. We used an open sky, daylight ambient illumination equivalent to CIE 6,500 K [73], that represent typical daylight conditions for foraging insects [74]. The transduction of photoreceptor captures (*P*) into receptor excitations (*E*) is given by

E=P/P+1. (3)

The receptor excitations (*E*_*UV*_, *E*_*B*_ *and E*_*G*_) are plotted on orthogonal axes, each of unit length, and the colour locus of a flower is the vector sum of the individual excitations. A colour locus can be represented by Cartesian coordinates in a hexagonal space using Eqs 4A and 4B [68]:

$$x=\sin(60{}^{\circ})(E_{G}-E_{UV})$$ (4A)

$$y=E_{B}-0.5(E_{G}-E_{UV}).$$ (4B)

Colour contrast in hexagon space was calculated as the Euclidean distance from the centre of the colour space, representing the adapting background, to the locus of a flower [75]. Hexagon coordinates for all flower species are given in online S1 Appendix.

## Data analysis

### Colour categories

For each species, we calculated the polar coordinates (angle and magnitude) of the floral colour locus in the hexagonal colour model (Fig 1A). The angle is a measurement of ‘hue’ in the hexagon space [68]. Samples were subsequently classified into one of the six colour categories proposed by [40] based on their respective hue value: BLUE (B), BLUE-GREEN (BG), GREEN (G), UV-GREEN (UG), UV (U), and UV-BLUE (UB).

### Does sugar content vary among colour categories?

To facilitate comparison with the results presented by [28], we classified flowers as having either a ‘high’ or ‘low’ soluble sugar content relative to the median for the entire flower sample. We used a set of contingency tables to test for significant differences in proportion of high and low soluble sugar content per color category, against a null hypothesis of equality of proportion per color group. Contingency tables excluded the single sample present in the UV color group (see results section for details). To avoid problems associated with using the χ^2^ distribution with small sample sizes in some colour categories, probability values from the chi-square test were obtained using 100,000 Monte Carlo simulations.

A second contingency test was conducted after excluding three species from the family Asteraceae because the soluble sugar content for these species was measured from a compound ‘head’ rather than individual flowers. Median soluble sugar amount was thus recalculated from the remaining species and the response variable was reformulated using the updated soluble sugar threshold value.

Finally, we constructed a third table excluding plant species in both Asteraceae and Orchidaceae, given the prevalence of potential food deception in many orchids [76–78]. As before, the median soluble sugar content was updated, and the remaining flower species were subsequently reclassified as being high or low.

### Correlation between sugar content and chromatic contrast

In addition to hue, chromatic contrast with a background (see *Colour space representation* above) is an element of colour that may be relevant to pollinators. We tested for a potential correlation between chromatic contrast of flower loci to the leaf green background in hexagon colour space and soluble sugar content. The analysis was done first on the entire data set, and subsequently for the two data subsets following the same rationale used for the contingency tests. All correlation tests were performed using Kendall’s tau (τ) statistic as the test makes no assumptions on the underlying distribution of the data [79]. All analyses were performed using R base package version 3.6.1 (05-07-2019).

#### Phylogenetic signal

**Fig 2:**
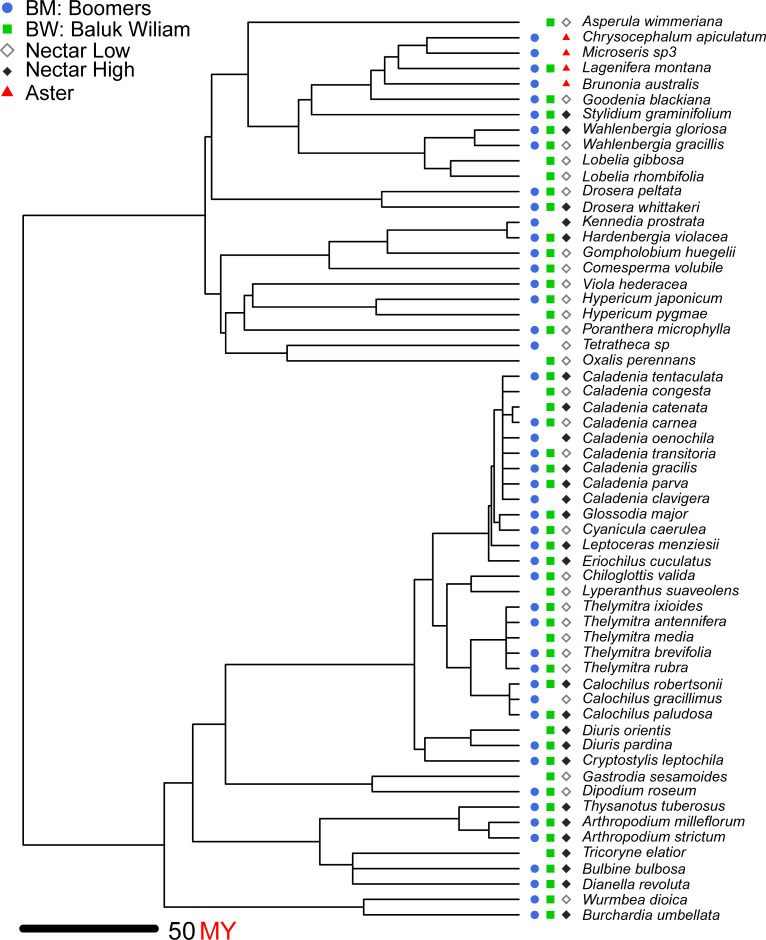
Phylogenetic tree showing the distribution of species at the BM (blue, solid circle) and BW (green, solid square) sites. Open squares indicate floral sugar content below the median value in the sample whereas solid square represents sugar content above the median value. These are the low and high categorizations used for comparison with the results in [28]. Red solid triangle shows the Aster group with higher sugar content then rest of the sample species. BM = Boomers Reserve, BW = Baluk Willam Flora reserve. S2 Appendix in supporting information S1 Table provides the nexus tree.

To estimate phylogenetic signal in the evolution of floral sugar content, we reconstructed the phylogeny of the species in our sample using the angiosperm family-level topology of Soltis et al. [80] as a scaffold and subfamilial topology from a variety of sources, and dated major nodes of the phylogenetic tree using the maximum-likelihood node ages from Wikström et al. [81] (Fig 2). We calculated phylogenetic signal in floral sugar content using Pagel’s λ [82] and tested its significance with a randomization technique implemented in the R package *phytools* [83]. For the entire data set, λ = 0.87, a value significantly greater than zero (*P* = 0.002) but similar to the value of unity expected under Brownian motion evolution of trait values. The apparent phylogenetic signal was, however, entirely due to the presence of species in the Asteraceae, for which sugar content of capitula rather than individual flowers was measured. Excluding the Asteraceae, there was no significant phylogenetic signal for floral sugar content (λ = 0.37, *P* > 0.05).

## Results

The distribution of species among hexagon sectors appeared uneven (Fig 1A and 1B), as did the distribution of soluble floral sugar per sector (Fig 1C). Only one sample was classified into the UV hexagon sector: *Hypericum pygmae* (Clusiaceae) and this hexagon sector was thus excluded from all subsequent analyses. The scarcity of flowers in UV sector is consistent with previous studies [40].

**Fig 3:**
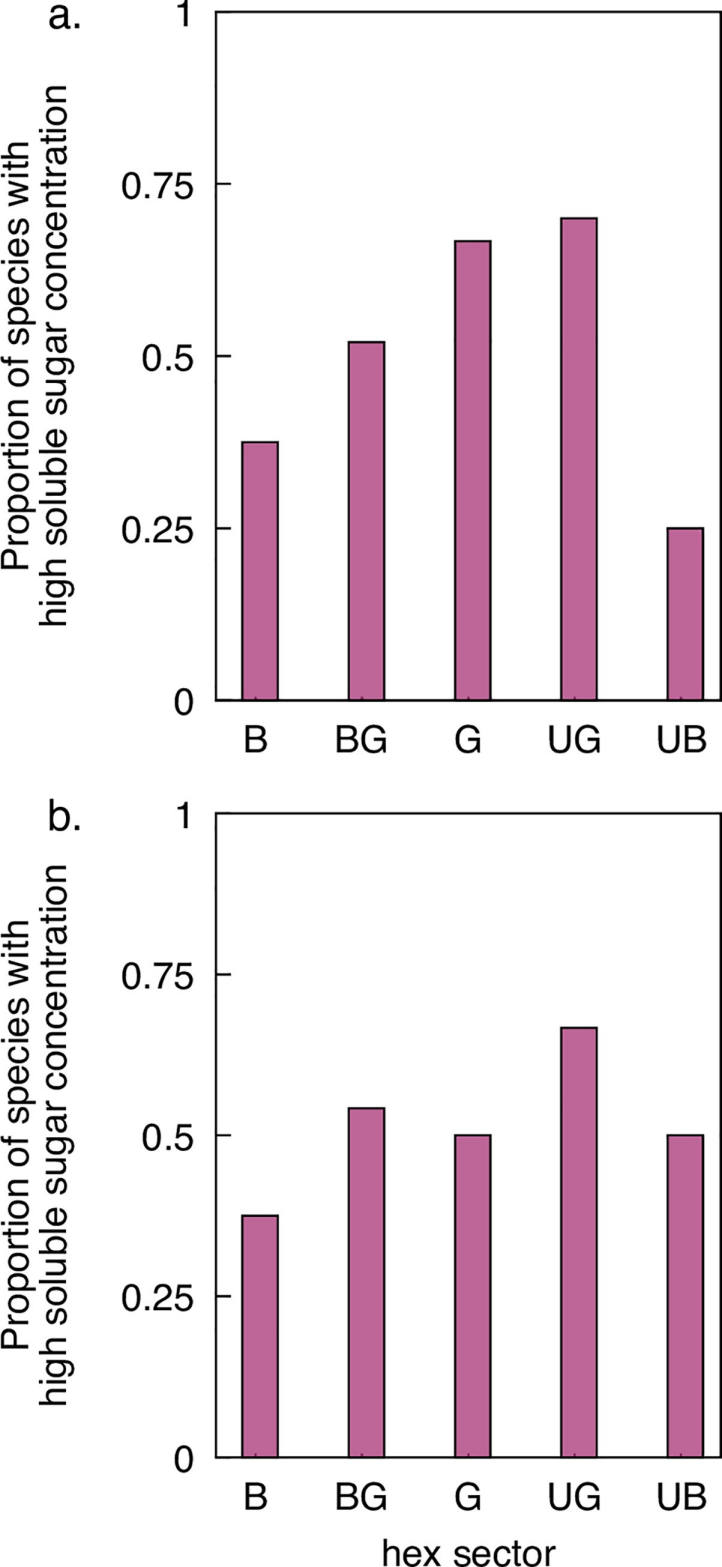
Proportion of species with a ‘high’ amount of soluble sugar for each one of the five categories including (panel a) and excluding species from the family Asteraceae (panel b).

Median soluble sugar content per flower ± median absolute deviation (MAD) for the sample excluding *Hypericum pygmae* was 392 ± 377 μg. Following categorization of species based on this threshold value (Fig 3A), we found no significant difference among hexagon sectors in the proportion of species with a high sugar content (χ^2^ = 3.97, *P* = 0.466).

Results from the second contingency table (median sugar soluble sugar = 383 ± 359 μg), which excluded species from the family Asteraceae also failed to reject the null hypothesis of equality in the proportion of species with a high amount of soluble sugar (χ^2^ = 2.15, *P* = 0.765, Fig 3B).

Finally, the threshold soluble sugar value for non-orchid, non-aster species contingency table was 367 ± 406 μg. For the third model we also found no evidence rejecting the null hypothesis of equality in the proportion of high-reward species among the different colour categories (χ^2^ = 2.97, *P* = 0.693). This result thus suggests that the low amount of soluble sugars present in orchid species at our field site was not a factor affecting a potentially the outcome of the initial model.

Chromatic contrast revealed no significant relation to floral sugar content at our field site. Tests using Kendall’s tau (τ) statistic failed to reject the null hypothesis of independence between soluble nectar content and chromatic contrast for all the subsets considered (Table 1 and also see S1 Table).

**Table 1:** Results of correlation analyses testing for a potential relation between soluble sugar content and chromatic contrast considering various sample subsets: Complete data set includes all flowers present at the two sampled locations. Following subsets sequentially remove species from the original data set: subset 1 (SS 1) excludes data from only species allocated to the UV hexagon sector *Hypericum pygmae*, subset two (SS 2) excludes species from the family Asteraceae from SS 1 and subset three (SS 3) excludes members of the family Orchidaceae from SS 2. Orchids were analysed separately in another subgroup (SS Orchids). A non-parametric (Kendall tau (τ)) correlation coefficient was calculated in all cases.

Data set	Sample size, *N*	τ	P
Complete data set	59	-0.016	0.86
Excluding flower in UV sector (SS 1)	58	-0.019	0.83
Excluding Asteraceae (SS 2)	55	-0.059	0.528
Excluding orchids (SS 3)	27	0.003	0.999
Only Orchidaceae (SS Orchids)	28	0.085	0.544

## Discussion

It was hypothesised by Darwin [84] that insects may evolve innate preferences to aid the efficient location of profitable flowers, and bees do have both innate spatial [5,85] and colour [28,34–39] preferences. In Germany, where important bee species like honeybees [28,34,36] and bumblebees [35,38,39] have innate preferences for short wavelength blue flowers, 80% of UV-BLUE flowers and just under 50% of BLUE flowers were found to be highly rewarding [28]. In contrast, UV-BLUE flowers at our Australian site were less likely than flowers in other colour categories to be highly rewarding (Fig 3A). Only 5% of UV-GREEN flowers in the German sample were highly rewarding [28], but 70% at our site (Fig 3A). Comparison between the sites is only approximate because the quantitative basis, if any, for distinguishing high- and low-reward species in the German sample was not explicitly made [28], while our division is at the median reward level. Nonetheless, it seems clear that the patterns of association of reward and floral colour are very dissimilar at the two sites.

Several studies suggest that nectar volume can influence the behaviour of foraging bees [11,12,19,20,86]. Indeed, it has also been shown that introduced species with flowers that contain higher volume rewards can out-compete resident flowers by attracting more bee pollinators [87], and in Mediterranean scrubland flowering community there was evidence that nectar-offering flowers had more chromatic contrast in a bee colour space compared to nectarless flower species [3]. Nonetheless, flower colours in different parts of the world have very similar distributions in bee colour space [45], which is also consistent with evidence that bees have phylogenetically conserved colour visual systems including innate preferences for short wavelength stimuli [28,30,35–39,41–43]. Thus, understanding whether flowers in different communities have colours that predict higher reward levels in a consistent fashion is of value for understanding what traits promote bee choices, and the potential major drivers that influence flower signal evolution in a particular environment.

In the current study we considered flower colour signaling from two communities in south-eastern Australia that had similar flower colours in bee colour space, and were also similar to bee pollinated flower colours found elsewhere across Australia and around the world [3,43,44,56,45]. We found no evidence that a particular hue predicted a higher reward level within flowers. These results from native plant communities suggest that a simple, direct link between flower rewards and the preferred bee hue of ‘blue’ is not an explanation of flower colour preferences in Australia. We also did not find a significant relationship between the chromatic contrast of a flower in hexagon colour space and the available nectar reward (Table 1). This is different to the significant relationship reported for Mediterranean scrubland, suggesting that different locations may yield different results depending upon environmental conditions [3].

Given the evidence that bee colour preferences may influence how flowers evolve similar spectral signals at several different locations around the world [40,43,44,45,57,88], it is interesting to consider what traits other than nectar rewards might promote bee preferences. Plausible alternative lines of investigation could include how the spectral overlap of photoreceptors when combined with opponent processes at a neural level enhance both colour discrimination [56,89–91] and colour detection [92] in a way that is most efficient for finding flowers [93]. This in turn could enhance neural mechanisms to promote innate colour preferences. By itself, this mechanism of spectral tuning cannot be the sole explanation for a stronger blue preference, since there is also spectral overlap and enhanced signal processing at longer wavelengths [56,89]. However, many common background stimuli reflect at longer wavelengths [94], so having innate brain preferences for shorter wavelength ‘blue’ stimuli might enable bees to efficiently detect stimuli that have a very high probability of being a rewarding flower given that very few natural colours are blue. Interestingly, UV absorbing flowers that appear ‘white’ to humans are very common within this short wavelength range of preferred colours in bee colour space, and additionally have the advantage of having strong modulation of the long wavelength bee receptor that is implicated in enhancing signal detection at a distance [69,95–98].

To better understand the complexities of these multiple complex factors it is important to collect more flower data at a plant community level around the world, as well as continue to map the sensory capabilities of different pollinators [16,90].

## Supporting information

S1 Appendix Provides the data used in current analysis. The ‘.csv’ file includes hexagon x and y unit, sucrose amount (microgram) and pollination categorization.
(CSV)

S2 Appendix Nexus file of Phylogenetic tree. (DOCX)

S1 Table Results of correlation analyses testing for a potential relation between soluble sugar content and chromatic contrast considering two different locations within each species subsets. (DOCX)

## References

[1] Kevan PG, Lane MA. Flower petal microtexture is a tactile cue for bees. Proc Natl Acad Sci. 1985;82: 4750–4752. Available: http://www.pnas.org/content/82/14/4750.abstract 10.1073/pnas.82.14.4750 16593582 PMC390982

[2] Leonard A, Masek P. Multisensory integration of colors and scents: insights from bees and flowers. J Comp Physiol A. Springer Berlin Heidelberg; 2014; 1–12. 10.1007/s00359-014-0904-4 24710696

[3] Kantsa A, Raguso RA, Dyer AG, Sgardelis SP, Olesen JM, Petanidou T. Community-wide integration of floral colour and scent in a Mediterranean scrubland. Nat Ecol Evol. 2017;1: 1502–1510. 10.1038/s41559-017-0298-0 29185514

[4] Shrestha M, Garcia JE, Bukovac Z, Dorin A, Dyer AG. Pollination in a new climate: Assessing the potential influence of flower temperature variation on insect pollinator behaviour. PLoS One. Public Library of Science; 2018;13: e0200549 Available: 10.1371/journal.pone.0200549 PMC6070230 30067757

[5] Howard S, Shrestha M, Schramme J, Garcia JE, Avarguès A, Greentree AD, et al Honeybee preferences and innate learning of flower achromatic morphologies. Curr Zool. 2019;65: 457–465. 10.1093/cz/zoy095 31413718 PMC6688580

[6] Barth FG. Insects and flowers. The biology of a partnership. Princeton: Princeton University Press USA.; 1985.

[7] PG K, Backhouse WG. Color vision: Ecology and evolution in making the best of the photic environment. Color Vision: Perspectives from Different Disciplines. 1998 pp. 163–183.

[8] Chittka L., Spaethe J., Schmidt A., Hickelsberger A. Adaptation, constraint, and chance in the evolution of flower color and pollinator color vision. In: Chittka L, Thomson JD(eds) Cognitive ecology of pollination Cambridge University Press, Cambridge, pp106–126. 2001;

[9] Fenster CB, Armbruster WS, Wilson P, Dudash MR, Thomson JD. Pollination syndromes and floral specialization. Annu Rev Ecol Evol Syst. 2004;35: 375–403. 10.1146/annurev.ecolsys.34.011802.132347

[10] Rausher Mark D., Rausher MD. Evolutionary transitions in floral color. Int J Plant Sci. The University of Chicago Press; 2008;169: 7–21. 10.1086/523358

[11] Waser NM, Price M V. The effect of nectar guides on pollinator preference: experimental studies with a montane herb. Oecologia. 1985;67: 121–126. 10.1007/BF00378462 28309856

[12] Willmer PG. The effects of insect visitors on nectar constituents in temperate plants. Oecologia. 1980;47: 270–277. 10.1007/BF00346832 28309483

[13] Stpiczyńska M, Nepi M, Zych M. Nectaries and male-biased nectar production in protandrous flowers of a perennial umbellifer Angelica sylvestris L. (Apiaceae). Plant Syst Evol. 2015;301: 1099–1113. 10.1007/s00606-014-1152-3

[14] Seeley TD, Camazine S, Sneyd J. Collective decision-making in honey bees: how colonies choose among nectar sources. Behav Ecol Sociobiol. Springer; 1991;28: 277–290.

[15] Nepi M, Grasso DA, Mancuso S. Nectar in plant–insect mutualistic relationships: From food reward to partner manipulation. Front Plant Sci. 2018;9: 1–14. 10.3389/fpls.2018.00001 30073014 PMC6060274

[16] Dyer AG, Paulk AC, Reser DH. Colour processing in complex environments: insights from the visual system of bees. Proc R Soc London B Biol Sci. The Royal Society; 2011;278: 952–959. 10.1098/rspb.2010.2412 PMC3049058 21147796

[17] Chalcoff VR, Gleiser G, Ezcurra C, Aizen MA. Pollinator type and secondarily climate are related to nectar sugar composition across the angiosperms. Evol Ecol. Springer International Publishing; 2017;31: 585–602. 10.1007/s10682-017-9887-2

[18] Heil M. Extrafloral Nectar at the Plant-Insect Interface: A Spotlight on Chemical Ecology, Phenotypic Plasticity, and Food Webs. Annu Rev Entomol. 2015;60: 213–232. 10.1146/annurev-ento-010814-020753 25564741

[19] van Rijn PCJ, Wäckers FL. Nectar accessibility determines fitness, flower choice and abundance of hoverflies that provide natural pest control. J Appl Ecol. 2016;53: 925–933. 10.1111/1365-2664.12605

[20] Gil M, De Marco RJ. Honeybees learn the sign and magnitude of reward variations. J Exp Biol. 2009;212: 2830–2834. 10.1242/jeb.032623 19684218

[21] Kemp DJ, Herberstein ME, Fleishman LJ, Endler JA, Bennett ATD, Dyer AG, et al An integrative framework for the appraisal of coloration in nature. Am Nat. University of Chicago Press Chicago, IL; 2015;185: 705–724. 10.1086/681021 25996857

[22] Knight TM, Steets JA, Vamosi JC, Mazer SJ, Burd M, Campbell DR, et al Pollen limitation of plant reproduction: Pattern and process. Annu Rev Ecol Evol Syst. Annual Reviews; 2005;36: 467–497. 10.1146/annurev.ecolsys.36.102403.115320

[23] Chittka L, Thomson JD, Waser NM. Flower constancy, insect psychology, and plant evolution. Naturwissenschaften. Springer-Verlag; 1999;86: 361–377. 10.1007/s001140050636

[24] Dyer AG, Chittka L. Biological significance of distinguishing between similar colours in spectrally variable illumination: bumblebees (Bombus terrestris) as a case study. J Comp Physiol A. Springer; 2004;190: 105–114. 10.1007/s00359-003-0475-2 14652688

[25] Burns JG, Dyer AG. Diversity of speed-accuracy strategies benefits social insects. Curr Biol. Cell Press; 2008;18: R953–R954. Available: http://linkinghub.elsevier.com/retrieve/pii/S0960982208011056 10.1016/j.cub.2008.08.028 18957249

[26] Bukovac Z, Dorin A, Dyer A. A-Bees See: A Simulation to Assess Social Bee Visual Attention During Complex Search Tasks. 2013; 276–283. 10.7551/978-0-262-31709-2-ch042

[27] Seeley TD. The wisdom of the hive: the social physiology of honey bee colonies. Harvard University Press; 2009.

[28] Giurfa M, Núñez J, Chittka L, Menzel R. Colour preferences of flower-naive honeybees. J Comp Physiol A. Springer-Verlag; 1995;177: 247–259. 10.1007/bf00192415

[29] Jacobs GH. Photopigments and the dimensionality of animal color vision. Neurosci Biobehav Rev. Elsevier; 2018;86: 108–130. 10.1016/j.neubiorev.2017.12.006 29224775

[30] Briscoe AD, Chittka L. The evolution of color vision in insects. Annu Rev Entomol. Annual Reviews 4139 El Camino Way, PO Box 10139, Palo Alto, CA 94303–0139, USA; 2001;46: 471–510. 10.1146/annurev.ento.46.1.471 11112177

[31] Kien J, Menzel R. Chromatic properties of interneurons in the optic lobes of the bee. J Comp Physiol. 1977;113: 17–34.

[32] Yang E-C, Lin H-C, Hung Y-S. Patterns of chromatic information processing in the lobula of the honeybee, Apis mellifera L. J Insect Physiol. 2004;50: 913–925. 10.1016/j.jinsphys.2004.06.010 15518659

[33] Paulk AC, Gronenberg W. Higher order visual input to the mushroom bodies in the bee, Bombus impatiens. Arthropod Struct Dev. 2008;37: 443–458. 10.1016/j.asd.2008.03.002 18635397 PMC2571118

[34] Menzel R. Untersuchungen zum erlernen von spektralfarben durch die honigbiene (Apis mellifica). Z Vgl Physiol. Springer-Verlag; 1967;56: 22–62. 10.1007/BF00333562

[35] Gumbert A. Color choices by bumble bees (Bombus terrestris): innate preferences and generalization after learning. Behav Ecol Sociobiol. Springer-Verlag; 2000;48: 36–43. 10.1007/s002650000213

[36] Morawetz L, Svoboda A, Spaethe J, Dyer AG. Blue colour preference in honeybees distracts visual attention for learning closed shapes. J Comp Physiol A. Springer; 2013;199: 817–827. 10.1007/s00359-013-0843-5 23918312

[37] Raine NE, Ings TC, Dornhaus A, Saleh N, Chittka L. Adaptation, genetic grift, pleiotropy, and history in the evolution of bee foraging behavior. Adv Study Behav. Elsevier; 2006;36: 305–354. 10.1016/S0065-3454(06)36007-X

[38] Raine NE, Chittka L. Colour preferences in relation to the foraging performance and fitness of the bumblebee Bombus terrestris. Uludag Bee J. 2005;5: 145–150.

[39] Raine NE, Chittka L. The adaptive significance of sensory bias in a foraging context: floral colour preferences in the bumblebee Bombus terrestris. PLoS One. Public Library of Science; 2007;2: e556 10.1371/journal.pone.0000556 17579727 PMC1891088

[40] Chittka L, Shmida A, Troje N, Menzel R. UV as a component of flower reflections and the colour perception of hymenoptera. Vision Res. 1994;34: 1489–1508. 10.1016/0042-6989(94)90151-1 8023461

[41] Dyer AG, Boyd-Gerny S, Shrestha M, Lunau K, Garcia JE, Koethe S, et al Innate colour preferences of the Australian native stingless bee Tetragonula carbonaria Sm. J Comp Physiol A. Springer; 2016;202: 603–613. 10.1007/s00359-016-1101-4 27316718

[42] Dyer AG, Boyd-Gerny S, Shrestha M, Garcia JE, van der Kooi CJ, Wong BBMM. Colour preferences of Tetragonula carbonaria Sm. stingless bees for colour morphs of the Australian native orchid Caladenia carnea. J Comp Physiol A. Springer Berlin Heidelberg; 2019;205: 347–361. 10.1007/s00359-019-01346-0 31139919

[43] Shrestha M, Dyer AG, Garcia JE, Burd M. Floral colour structure in two Australian herbaceous communities: it depends on who is looking. Ann Bot. 2019;124: 221–232. 10.1093/aob/mcz043 31008511 PMC6758583

[44] Shrestha M, Dyer AG, Bhattarai P, Burd M. Flower colour and phylogeny along an altitudinal gradient in the Himalayas of Nepal. J Ecol. 2014;102: 126–135. 10.1111/1365-2745.12185

[45] Dyer AG, Boyd-Gerny S, McLoughlin S, Rosa MGP, Simonov V, Wong BBM. Parallel evolution of angiosperm colour signals: common evolutionary pressures linked to hymenopteran vision. Proc R Soc B. The Royal Society; 2012;279: 3606–3615. Available: http://rspb.royalsocietypublishing.org/content/279/1742/3606.abstract 10.1098/rspb.2012.0827 PMC3396912 22673351

[46] Arnold SJ, Savolainen V, Chittka L. Flower colours along an alpine altitude gradient, seen through the eyes of fly and bee pollinators. Arthropod Plant Interact. Springer Netherlands; 2009;3: 27–43. 10.1007/s11829-009-9056-9

[47] McEwen JR, Vamosi JC. Floral colour versus phylogeny in structuring subalpine flowering communities. Proc R Soc B Biol Sci. 2010;277: 2957–2965. 10.1098/rspb.2010.0501 PMC2982023 20484236

[48] Ohashi K, Makino TT, Arikawa K. Floral colour change in the eyes of pollinators: testing possible constraints and correlated evolution. Funct Ecol. 2015;29: 1144–1155. 10.1111/1365-2435.12420

[49] Makino TT, Yokoyama J. Nonrandom composition of flower colors in a plant community: mutually different co-flowering natives and disturbance by aliens. PLoS One. Public Library of Science; 2015;10: e0143443 10.1371/journal.pone.0143443 26650121 PMC4674055

[50] Kantsa A, Raguso RA, Dyer AG, Olesen JM, Tscheulin T, Petanidou T. Disentangling the role of floral sensory stimuli in pollination networks. Nat Commun. 2018;9: 1041 10.1038/s41467-018-03448-w 29531220 PMC5847531

[51] Gray M, Stansberry MJ, Lynn JS, Williams CF, White TE, Whitney KD. Consistent shifts in pollinator-relevant floral coloration along Rocky Mountain elevation gradients. J Ecol. 2018;106: 1910–1924. 10.1111/1365-2745.12948

[52] Kemp JE, Ellis AG. Cryptic petal coloration decreases floral apparency and herbivory in nocturnally closing daisies. Funct Ecol. 2019; 1–12. 10.1111/1365-2435.13423

[53] de Camargo MGG, Lunau K, Batalha MA, Brings S, de Brito VLG, Morellato LPC. How flower colour signals allure bees and hummingbirds: a community-level test of the bee avoidance hypothesis. New Phytol. 2019;222: 1112–1122. 10.1111/nph.15594 30444536

[54] Kemp JE, Bergh NG, Soares M, Ellis AG. Dominant pollinators drive non-random community assembly and shared flower colour patterns in daisy communities. Ann Bot. 2018; mcy126–mcy126. Available: 10.1093/aob/mcy126 PMC6344215 29992277

[55] Shrestha M, Burd M, Garcia JE, Dorin A, Dyer AG. Colour evolution within orchids depends on whether the pollinator is a bee or a fly. Plant Biol. John Wiley & Sons, Ltd (10.1111); 2019;0. 10.1111/plb.12968 30681768

[56] Chittka L, Menzel R. The evolutionary adaptation of flower colours and the insect pollinators’ colour vision. J Comp Physiol A. Springer-Verlag; 1992;171: 171–181. 10.1007/BF00188925

[57] Bischoff M, Lord JM, Robertson AW, Dyer AG, M, Bischoff JM, Lord AW, et al Hymenopteran pollinators as agents of selection on flower colour in the New Zealand mountains: salient chromatic signals enhance flower discrimination. New Zeal J Bot. Taylor & Francis; 2013;51: 181–193. 10.1080/0028825X.2013.806933

[58] Shrestha M, Dyer AG, Boyd-gerny S, Wong BBM, Burd M. Shades of red: bird-pollinated flowers target the specific colour discrimination abilities of avian vision. New Phytol. 2013;198: 301–310. 10.1111/nph.12135 23368754

[59] Jeans J, Backhouse G. Wild Orchids of Victoria Australia. Published by Rudie H. Kuiter publisher 2006;

[60] Jones DL. A complete guide to Native Orchids of Australia. New Holland Publishers (Australia) Pty Ltd 2006;

[61] Richardson FJ, Richardson RJ, Shepherd RCH. Weeds of the South-East: an identification guide for Australia. Published by R.G. and F. J. Richardson, Australia (www.weedinfo.co.au). 2011;

[62] Ross JH. A census of the vascular plants of Victoria. Published by the National Herbarium of Victoria, Royal Botanical Gardens, Victoria, 3141, Australia. 2000;

[63] Corrick MG, Fuhrer BA. Wild flowers of Victoria and adjoining areas. Hawthorn, Vic: Published by Bloomings Book, Victoria, Australia.; 2008.

[64] Walsh NG, Entwisle TJ, W N.G., Walsh NG, Entwisle TJ. Flora of Victoria, vol 4 Inkata Press, Melbourne; 1999.

[65] Walsh NG, Entwisle TJ, W N.G., Walsh NG, Entwisle TJ. Flora of Victoria,vol.2 Inkata Press, Melbourne; 1994.

[66] Walsh NG, Entwisle TJ, W N.G., Walsh NG, Entwisle TJ. Flora of Victoria,vol.3 Inkata Press, Melbourne; 1996.

[67] Roberts RB. Spectrophotometric analysis of sugars produced by plants and harvested by insects. J Apic Res. Taylor & Francis; 1979;18: 191–195. 10.1080/00218839.1979.11099966

[68] Chittka L. The colour hexagon: a chromaticity diagram based on photoreceptor excitations as a generalized representation of colour opponency. J Comp Physiol A. Springer-Verlag; 1992;170: 533–543. 10.1007/BF00199331

[69] Dyer AG, Streinzer M, Garcia J. Flower detection and acuity of the Australian native stingless bee Tetragonula carbonaria Sm. J Comp Physiol A. 2016;202: 629–639. 10.1007/s00359-016-1107-y 27380933

[70] Whitney HM, Chittka L, Bruce TJA, Glover BJ. Conical epidermal cells allow bees to grip flowers and increase foraging efficiency. Curr Biol. 2009;19: 948–953. 10.1016/j.cub.2009.04.051 19446458

[71] Stavenga DG, Smits RP, Hoenders BJ. Simple exponential functions describing the absorbance bands of visual pigment spectra. Vision Res. 1993;33: 1011–1017. 10.1016/0042-6989(93)90237-q 8506642

[72] Endler JA. On the measurement and classification of colour in studies of animal colour patterns. Biol J Linn Soc. Blackwell Publishing Ltd; 1990;41: 315–352. 10.1111/j.1095-8312.1990.tb00839.x

[73] Judd DB, MacAdam DL, Wyszecki G, Budde HW, Condit HR, Henderson ST, et al Spectral Distribution of Typical Daylight as a Function of Correlated Color Temperature. J Opt Soc Am. OSA; 1964;54: 1031–1040. 10.1364/JOSA.54.001031

[74] Garcia JE, Spaethe J, Dyer AG. The path to colour discrimination is S-shaped: behaviour determines the interpretation of colour models. J Comp Physiol A Neuroethol Sensory, Neural, Behav Physiol. Springer Berlin Heidelberg; 2017;203: 983–997. 10.1007/s00359-017-1208-2 28866838

[75] Spaethe J, Tautz J, Chittka L. Visual constraints in foraging bumblebees: Flower size and color affect search time and flight behavior. Proc Natl Acad Sci. 2001;98: 3898–3903. 10.1073/pnas.071053098 11259668 PMC31150

[76] Mimicry Dafni A. and deception in pollination. Annu Rev Ecol Syst. 1984;15: 259–278. 10.1146/annurev.es.15.110184.001355

[77] Ackerman JD. Pollination of tropical and temperate orchids. Proceedings of the Eleventh World Orchid Conference Miami, Florida: American Orchid Society. 1985. pp. 98–101.

[78] Shrestha M, Dyer AG, Dorin A, Ren Z-X, Burd M. Rewardlessness in orchids: how frequent and how rewardless? Plant Biol. John Wiley & Sons, Ltd; 2020;n/a. 10.1111/plb.13113 32181557

[79] Zar JH. Biostatistical analysis. 4th ed Prentice Hall, New Jersey, USA; 1999.

[80] Soltis DE, Smith SA, Cellinese N, Wurdack KJ, Tank DC, Brockington SF, et al Angiosperm phylogeny: 17 genes, 640 taxa. Am J Bot. 2011;98: 704–730. 10.3732/ajb.1000404 21613169

[81] Wikström N, Savolainen V, Chase MW. Evolution of the angiosperms: calibrating the family tree. Proc R Soc London B Biol Sci. 2001;268: 2211–2220. Available: http://rspb.royalsocietypublishing.org/content/268/1482/2211.abstract 10.1098/rspb.2001.1782 PMC1088868 11674868

[82] Pagel M. Inferring the historical patterns of biological evolution. Nature. 1999;401: 877–884. Available: http://cat.inist.fr/?aModele=afficheN&cpsidt=1178332 10.1038/44766 10553904

[83] Revell LJ. Phytools: an R package for phylogenetic comparative biology (and other things). Methods Ecol Evol. Blackwell Publishing Ltd; 2012;3: 217–223. 10.1111/j.2041-210X.2011.00169.x

[84] Darwin C. The effects of cross and self fertilisation in the vegetable kingdom. J. Murray, London; 1877.

[85] Lehrer M, Horridge GA, Zhang SW, Gadagkar R. Shape vision in bees: Innate preference for flower-like patterns. Philos Trans R Soc B Biol Sci. 1995;347: 123–137. 10.1098/rstb.1995.0017

[86] Kunze J, Chittka L. Butterflies and bees fly faster when plants feed them more nectar. Goettingen Neurobiology Report. 1996 p. 109.

[87] Chittka L, Schürkens S. Successful invasion of a floral market. Nature. 2001;411: 2001. 10.1038/35079676 11395755

[88] Suzuki MF, Ohashi K. How does a floral colour-changing species differ from its non-colour-changing congener?–a comparison of trait combinations and their effects on pollination. Funct Ecol. John Wiley & Sons, Ltd (10.1111); 2014;28: 549–560. 10.1111/1365-2435.12209

[89] von Helversen O. Zur spektralen unterschiedsempfindlichkeit der honigbiene. J Comp Physiol. Springer; 1972;80: 439–472.

[90] Chittka L, Wells H. Color vision in bees: mechanisms, ecology and evolution. In: Prete F.: Complex Worlds from simpler nervous systems; MIT Press, Boston pp. 165–191. 2004;

[91] Peitsch D, Fietz A, Hertel H, de Souza J, Ventura DF, Menzel R. The spectral input systems of hymenopteran insects and their receptor-based colour vision. J Comp Physiol A. Springer; 1992;170: 23–40. 10.1007/BF00190398 1573568

[92] Bukovac Z, Dorin A, Finke V, Shrestha M, Garcia J, Avarguès-Weber A, et al Assessing the ecological significance of bee visual detection and colour discrimination on the evolution of flower colours. Evol Ecol. 2017;31: 153–172. 10.1007/s10682-016-9843-6

[93] Chittka L. Optimal sets of color receptors and color opponent systems for coding of natural objects in insect vision. J Theor Biol. 1996;181: 179–196. 10.1006/jtbi.1996.0124

[94] Bukovac Z, Shrestha M, Garcia JE, Burd M, Dorin A, Dyer AG. Why background colour matters to bees and flowers. J Comp Physiol A. 2017;203: 1–12. 10.1007/s00359-017-1175-7 28478535

[95] Giurfa M, Vorobyev M, Kevan P, Menzel R. Detection of coloured stimuli by honeybees: Minimum visual angles and receptor specific contrasts. J Comp Physiol A. Springer-Verlag; 1996;178: 699–709. 10.1007/BF00227381

[96] Stach S, Benard J, Giurfa M. Local-feature assembling in visual pattern recognition and generalization in honeybees. Nature. 2004;429: 758–761. 10.1038/nature02594 15201910

[97] Dyer AG, Spaethe J, Prack S. Comparative psychophysics of bumblebee and honeybee colour discrimination and object detection. J Comp Physiol A. Springer-Verlag; 2008;194: 617–627. 10.1007/s00359-008-0335-1 18437390

[98] Avarguès-Weber A, Giurfa M. Cognitive components of color vision in honey bees: how conditioning variables modulate color learning and discrimination. J Comp Physiol A. 2014;200: 449–461. 10.1007/s00359-014-0909-z 24788332

