# Supplementary material for: Australian native flower colours: Does nectar reward drive bee pollinator flower preferences?
Source: PLoS One. 2020 Jun 11;15(6):e0226469. doi: 10.1371/journal.pone.0226469 (PMC7289428; doi:10.1371/journal.pone.0226469)
Supplement: S2 Appendix — (DOCX) [file pone.0226469.s002.docx]

Appendix B: Phylogenetic tree

BEGIN TAXA;

TITLE Taxa;

DIMENSIONS NTAX=59;

TAXLABELS

Burchardia_umbellata Wurmbea_dioica Tricoryne_elatior Dianella_revoluta Bulbine_bulbosa Arthropodium_strictum Thysanotus_tuberosus Arthropodium_milleflorum Leptoceras_menziesii Gastrodia_sesamoides Dipodium_roseum Cryptostylis_leptochila Diuris_pardina Diuris_orientis Calochilus_paludosa Calochilus_gracillimus Calochilus_robertsonii Thelymitra_rubra Thelymitra_media Thelymitra_brevifolia Thelymitra_antennifera Thelymitra_ixioides Lyperanthus_suaveolens Chiloglottis_valida Eriochilus_cuculatus Cyanicula_caerulea Glossodia_major Caladenia_transitoria Caladenia_tentaculata Caladenia_parva Caladenia_oenochila Caladenia_gracilis Caladenia_congesta Caladenia_clavigera Caladenia_carnea Caladenia_catenata Oxalis_perennans Tetratheca_sp Poranthera_microphylla Hypericum_pygmae Hypericum_japonicum Viola_hederacea Comesperma_volubile Gompholobium_huegelii Hardenbergia_violacea Kennedia_prostrata Drosera_whittakeri Drosera_peltata Lobelia_rhombifolia Lobelia_gibbosa Wahlenbergia_gracillis Wahlenbergia_gloriosa Stylidium_graminifolium Brunonia_australis Goodenia_blackiana Lagenifera_montana Microseris_sp3 Chrysocephalum_apiculatum Asperula_wimmeriana

;

END;

BEGIN TREES;

Title 'Trees from "Nectar_R_simulated_tree_07April20.nex"';

ID 017151c489e21;

LINK Taxa = Taxa;

TRANSLATE

[0] 1 Burchardia_umbellata,

[1] 2 Wurmbea_dioica,

[2] 3 Tricoryne_elatior,

[3] 4 Dianella_revoluta,

[4] 5 Bulbine_bulbosa,

[5] 6 Arthropodium_strictum,

[6] 7 Thysanotus_tuberosus,

[7] 8 Arthropodium_milleflorum,

[8] 9 Leptoceras_menziesii,

[9] 10 Gastrodia_sesamoides,

[10] 11 Dipodium_roseum,

[11] 12 Cryptostylis_leptochila,

[12] 13 Diuris_pardina,

[13] 14 Diuris_orientis,

[14] 15 Calochilus_paludosa,

[15] 16 Calochilus_gracillimus,

[16] 17 Calochilus_robertsonii,

[17] 18 Thelymitra_rubra,

[18] 19 Thelymitra_media,

[19] 20 Thelymitra_brevifolia,

[20] 21 Thelymitra_antennifera,

[21] 22 Thelymitra_ixioides,

[22] 23 Lyperanthus_suaveolens,

[23] 24 Chiloglottis_valida,

[24] 25 Eriochilus_cuculatus,

[25] 26 Cyanicula_caerulea,

[26] 27 Glossodia_major,

[27] 28 Caladenia_transitoria,

[28] 29 Caladenia_tentaculata,

[29] 30 Caladenia_parva,

[30] 31 Caladenia_oenochila,

[31] 32 Caladenia_gracilis,

[32] 33 Caladenia_congesta,

[33] 34 Caladenia_clavigera,

[34] 35 Caladenia_carnea,

[35] 36 Caladenia_catenata,

[36] 37 Oxalis_perennans,

[37] 38 Tetratheca_sp,

[38] 39 Poranthera_microphylla,

[39] 40 Hypericum_pygmae,

[40] 41 Hypericum_japonicum,

[41] 42 Viola_hederacea,

[42] 43 Comesperma_volubile,

[43] 44 Gompholobium_huegelii,

[44] 45 Hardenbergia_violacea,

[45] 46 Kennedia_prostrata,

[46] 47 Drosera_whittakeri,

[47] 48 Drosera_peltata,

[48] 49 Lobelia_rhombifolia,

[49] 50 Lobelia_gibbosa,

[50] 51 Wahlenbergia_gracillis,

[51] 52 Wahlenbergia_gloriosa,

[52] 53 Stylidium_graminifolium,

[53] 54 Brunonia_australis,

[54] 55 Goodenia_blackiana,

[55] 56 Lagenifera_montana,

[56] 57 Microseris_sp3,

[57] 58 Chrysocephalum_apiculatum,

[58] 59 Asperula_wimmeriana;

TREE UNTITLED = (((1:58.9522,2:58.9522):75.76081,(((3:63.13079,4:63.13079,5:63.13079)Xanthorrhoeaceae:12.49512,((6:11.37828,8:11.37828):11.37828,7:22.75656):52.86935):48.23824,((11:55.722505,10:55.722505):55.722505,((12:35.68116,(13:18.26992,14:18.26992):17.41126):4.01231,((((15:3.58718,16:3.58718,17:3.58718):14.70379,(18:4.8153,19:4.8153,20:4.8153,21:4.8153,22:4.81529):13.47568):9.05908,(23:18.1446,24:18.1446):9.20544):5.35729,(25:11.48038,(9:10.441765,((26:7.29126,27:7.29127):2.11189,(28:6.18179,29:6.18179,30:6.18179,31:6.18179,32:6.18179,33:6.18179,34:6.18179,(35:2.42337,36:2.42337):3.75841):3.22137):1.038615):1.038615):21.22694):6.98614):71.75152)Orchidaceae:12.41914):10.84887):53.57023,((((37:88.04912,38:88.04912):23.3319,(39:104.29499,((40:54.21777,41:54.21778):46.89628,42:101.11405):3.18095):7.08601):1.87759,(43:72.04879,(44:49.95386,(45:4.48923,46:4.48922):45.46463):22.09495)Fabales:41.20982)Fabidae:5.9069,((47:52.09178,48:52.09178):64.6055,((((49:25.90274,50:25.90274):9.91275,(51:16.79797,52:16.79796):19.01752):47.84818,(53:78.73798,(55:56.19418,(54:50.96894,(56:45.7437,(57:34.94052,58:34.94052):10.8032)Asteraceae:5.22524):5.22524):22.54381):4.92568)Asterales:19.20781,59:102.8715):13.82579)Pentapetalae:2.46824)Gunneridae:69.11774)[% ] [% ] [% setBetweenBits = selected ];

END;
