# Supplementary material for: Australian native flower colours: Does nectar reward drive bee pollinator flower preferences?
Source: PLoS One. 2020 Jun 11;15(6):e0226469. doi: 10.1371/journal.pone.0226469 (PMC7289428; doi:10.1371/journal.pone.0226469)
Supplement: S1 Table — (DOCX) [file pone.0226469.s003.docx]

**Table S1:** Results of correlation analyses testing for a potential relation between soluble sugar content and chromatic contrast considering two different locations within each species subsets. Complete data set includes all flowers present at the two sampled locations: Boomers Reserve (BM) and Baluk Willam reserve (BW). Subsets sequentially remove species from the original data set: subset 1 (SS 1) excludes data from only species allocated to the UV hexagon sector *Hypericum pygmae*, subset two (SS 2) excludes species from the family Asteraceae from SS 1 and subset three (SS 3) excludes members of the family Orchidaceae from SS 2. Orchids were analysed separately in another subgroup (SS Orchids). A non-parametric (Kendall tau (τ)) correlation coefficient was calculated in all cases.

| Subset | Location | *N* | τ | P-value |
| --- | --- | --- | --- | --- |
| All species | BM | 47 | 0.025 | 0.813 |
|  | BW | 51 | 0.013 | 0.890 |
| Excluding UV (SS 1) | BM | 47 | 0.025 | 0.813 |
|  | BW | 50 | 0.010 | 0.920 |
| Excluding Asteraceae (SS 2) | BM | 44 | -0.017 | 0.880 |
|  | BW | 49 | 0.017 | 0.871 |
| Excluding Orchidaceae (SS 3) | BM | 22 | 0.065 | 0.696 |
|  | BW | 24 | 0.109 | 0.476 |
| Orchidaceae (SS Orchids) | BM | 22 | -0.065 | 0.696 |
|  | BW | 25 | -0.027 | 0.871 |
